# Genetic Overlap Analysis Identifies a Shared Etiology between Migraine and Headache with Type 2 Diabetes

**DOI:** 10.3390/genes13101845

**Published:** 2022-10-12

**Authors:** Md Rafiqul Islam, The International Headache Genetics Consortium (IHGC), Dale R. Nyholt

**Affiliations:** Statistical and Genomic Epidemiology Laboratory, School of Biomedical Sciences, Faculty of Health, Centre for Genomics and Personalised Health, Queensland University of Technology, Kelvin Grove, QLD 4000, Australia

**Keywords:** migraine, headache, GWAS, type 2 diabetes, genetic correlation, shared genetics, Mendelian randomisation, genetic overlap, pleiotropy

## Abstract

Migraine and headache frequently co-occur with type 2 diabetes (T2D), suggesting a shared aetiology between the two conditions. We used genome-wide association study (GWAS) data to investigate the genetic overlap and causal relationship between migraine and headache with T2D. Using linkage disequilibrium score regression (LDSC), we found a significant genetic correlation between migraine and T2D (*r*_g_ = 0.06, *p* = 1.37 × 10^−5^) and between headache and T2D (*r*_g_ = 0.07, *p* = 3.0 × 10^−4^). Using pairwise GWAS (GWAS-PW) analysis, we identified 11 pleiotropic regions between migraine and T2D and 5 pleiotropic regions between headache and T2D. Cross-trait SNP meta-analysis identified 23 novel SNP loci (*P*_meta_ < 5 × 10^−8^) associated with migraine and T2D, and three novel SNP loci associated with headache and T2D. Cross-trait gene-based overlap analysis identified 33 genes significantly associated (*P*_gene-based_ < 3.85 × 10^−6^) with migraine and T2D, and 11 genes associated with headache and T2D, with 7 genes (*EHMT2*, *SLC44A4*, *PLEKHA1*, *CFDP1*, *TMEM170A*, *CHST6*, and *BCAR1*) common between them. There was also a significant overlap of genes nominally associated (*P*_gene-based_ < 0.05) with both migraine and T2D (*P*_binomial-test_ = 2.83 × 10^−46^) and headache and T2D (*P*_binomial-test_ = 4.08 × 10^−29^). Mendelian randomisation (MR) analyses did not provide consistent evidence for a causal relationship between migraine and T2D. However, we found headache was causally associated (inverse-variance weighted, OR_IVW_ = 0.90, *P*_ivw_ = 7 × 10^−3^) with T2D. Our findings robustly confirm the comorbidity of migraine and headache with T2D, with shared genetically controlled biological mechanisms contributing to their co-occurrence, and evidence for a causal relationship between headache and T2D.

## 1. Introduction

Migraine is a chronic recurrent neurological condition, typically characterised by repeated attacks of headache lasting between 4 and 72 h, often accompanied by nausea, vomiting, photophobia, and phonophobia, that affects up to 14.7% of the world’s population and is the second leading cause of disability [1]. The vascular system has long been considered to play a role in migraine pathophysiology. GWAS have recently provided evidence for vascular involvement in migraine [2,3]. The association between migraine and the vascular system is supported by several physiologic and observational studies, including migraine comorbidity with other vascular traits, such as blood pressure [4], stroke [5], and coronary artery disease [6]. T2D is a well-established risk factor for vascular disease [7] and observational studies have found that people with migraine and headaches are more likely to develop T2D than the general population [8,9]. Some studies have suggested that migraine and headaches increase the risk of T2D, while others have found an inverse or no association between them [10,11]. Additionally, a recent cross-trait GWAS reported an association between migraine and T2D [12]. Despite the epidemiological evidence, there are still many unanswered questions regarding the relationship between migraine and headaches with T2D. T2D is a long-term risk factor for various debilitating and potentially life-threatening complications. T2D affects an estimated 462 million people worldwide, accounting for 6.28% of the world’s population, imposing huge burdens on individuals and healthcare management systems [13]. Migraine, headache, and T2D are common complex traits with multiple aetiologic contributors [14,15]. Among these, genetic predisposition factors play a significant role in all these conditions. In combination with environmental factors, multiple genetic factors influence susceptibility to such complex traits. A strong genetic base for each trait has been discovered [2,16,17]. However, whether there is a shared or interactive genetic background behind all three traits remains largely unknown. We hypothesise that T2D may share underlying genetic aetiologies with migraine and headaches. The existence of these shared genetic aetiologies between these traits in a subset of people may lead to migraine and headaches and the long-term development of T2D.

GWAS has been successfully used to identify genetic risk variants for complex traits, in which hundreds of thousands to millions of single nucleotide polymorphisms (SNPs) are genotyped and tested for association [18]. Several GWAS studies in migraine, headache, and T2D have been published, some of which have been robustly replicated in large sample sizes [2,16,17]. This increasing number of GWAS studies allows combining recent results from various investigations to improve statistical power and identify regions and variants with small effects. Furthermore, combining data from multiple interactive traits may uncover underlying shared genetic mechanisms. The growing availability of GWAS datasets has fuelled the development of methods to investigate the shared genetic architecture and casual genetic relationships between traits [18]. The current study used multiple GWAS methods to determine whether migraine and headaches share similar genetic etiological factors with T2D and, if so, to decipher the potential impacts of these genetic factors on cellular or molecular pathways, leading to the development of migraine, headache, and T2D. Our results provide a deeper understanding of the relationship between migraine, headache, and T2D. This knowledge is of primary importance in directing efforts towards possible therapeutic strategies in a particular migraine subpopulation and the long-term management of migraine and T2D.

In addition, several epidemiological observational studies have shown that women have a substantially higher risk of migraine than men [19,20]; however, the sex difference in migraine risk in diabetic patients is currently ambiguous. Therefore, we used combined and sex-stratified GWAS summary statistics for migraine, headache, and T2D from different sources (i.e., International headache genetic consortium (IHGC), Diabetes genetics replication and meta-analysis consortium (DIAGRAM), and UK Biobank (UKB) Neale Lab) to explore the genetic association between migraine and headache with T2D in Europeans.

## 2. Materials and Methods

### 2.1. Study Population and Design

**Figure 1:**
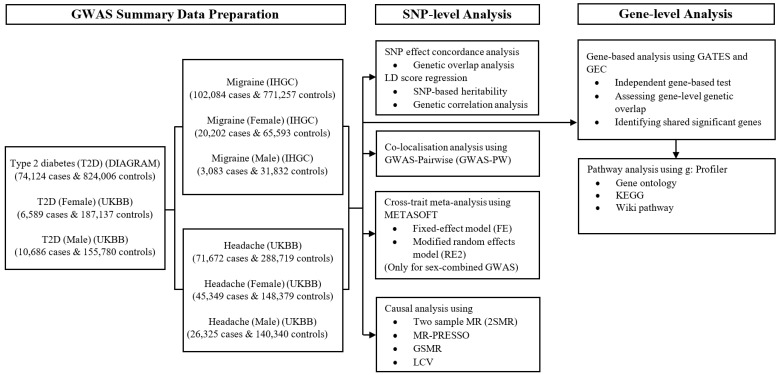
Overall study design and outline of the analysis. IHGC, International headache genetic consortium; DIAGRAM, Diabetes genetics replication and meta-analysis consortium; UKBB, UK Biobank (Neale Lab); PPA, Posterior probabilities of association; LD, Linkage disequilibrium; GATES, Gene-Based Association Test Using Extended Simes Procedure; GEC, Genetic type 1 error calculator; GWAS, Genome-wide association study; KEGG, Kyoto Encyclopedia of Genes and Genomes; LCV, Latent causal variable; MR, Mendelian randomisation; MR-PRESSO, Mendelian randomisation pleiotropy residual sum and outlier; GSMR, Generalised summary data-based Mendelian Randomisation; SNP, Single-nucleotide polymorphism.

The overall study design (summarised in Figure 1) included three key data sources: the IHGC, UK Biobank, and DIAGRAM consortiums. We used combined and sex-stratified GWAS summary statistics for migraine and broadly defined headache (experienced last month), including data from the IHGC and the UKB sample analysed by the Neale group, respectively. In addition, GWAS summary statistics for T2D (combined) were obtained from the DIAGRAM consortium, and sex-specific T2D were included from the UKB (Neale Lab).

### 2.2. Summary Statistics for Migraine, Headache, and T2D

The genetic overlap analyses utilised the largest available GWAS summary statistics. The migraine GWAS summary statistics were obtained from a GWAS of 102,084 cases and 771,257 controls of European descent [2]. More information about data collection methods and techniques can be found in the original publication [2]. The T2D GWAS summary statistics were obtained from the DIAGRAM consortium (https://www.diagram-consortium.org/ (accessed on 10 August 2019)) from a T2D GWAS of 74,124 cases and 824,006 controls [17]. The headache GWAS summary statistics were obtained from the UKB GWAS for ‘headache pain experienced last month’ (UKB data field: 6159_1), comprising 71,672 cases and 288,719 controls, which are publicly accessible at http://www.nealelab.is/uk-biobank/ (accessed on 15 March 2021) [21]. Headache cases in our study were those who experienced a headache in the last month that interfered with their usual activities [21]. Detailed phenotypic descriptions for headache GWAS are available at the following link (https://biobank.ctsu.ox.ac.uk/crystal/field.cgi?id=6159 (accessed on 15 March 2021)).

According to the International Headache Society, headaches are classified into primary and secondary headaches [22]. Migraine, tension-type, and cluster headaches are all examples of primary headaches. In contrast, secondary headaches are associated with certain medical conditions such as head injury, neoplasm, and head pain caused by infection [22]. Given over 90% of migraine sufferers also have tension-type headaches, and ~50% of genetic risk loci for headaches overlapped with previously reported migraine loci [16], we also examined the relationship between headache and T2D to identify genetic variants associated with headache and compare these findings to the analysis of migraine and T2D.

### 2.3. Summary Statistics for Sex-Stratified Migraine, Headache, and T2D

We analysed GWAS sex-stratified summary statistics for migraine from the study of Anttila et al. (2013), which analysed a total of 23,285 cases and 95,425 controls, of which 3083 cases and 31,832 controls were male; and 20,202 cases and 63,593 controls were female, obtained from the IHGC (http://www.headachegenetics.org/ (accessed on 15 March 2020)) [23]. From the Neale Lab (http://www.nealelab.is/uk-biobank/ (accessed on 15 March 2021)) we downloaded UKB sex-stratified GWAS summary statistics for T2D, of which 10,686 cases and 155,780 controls were male, and 6589 cases and 187,137 controls were female; and headache, of which 26,324 cases and 140,340 controls were male and 45,348 cases and 148,379 controls were female. UKB data field 2443 was used to determine doctor-diagnosed diabetes, and data field 6159_1 was used to determine headache pain experienced last month for sex-specific GWAS data. These GWAS studies were conducted on individuals of European ancestry.

### 2.4. Analysis of Sex-Stratified Effects

Previous studies have demonstrated that the association between migraine and headache with T2D differs by sex [10], and females are at increased risk for migraine [24]. In addition, migraine and headaches are more prevalent in females than males, and sex hormones, particularly oestrogen and progesterone fluctuations, are thought to play an important role [11,25]. Therefore, we conducted sex-specific analyses to determine if the genetic relationship between migraine and headache with T2D differs by sex.

### 2.5. Pre-Processing of GWAS Data

Where available, we obtained missing rsIDs for SNPs using dbSNP151 (http://www.ncbi.nlm.nih.gov/SNP (accessed on 10 December 2020)). We dropped SNPs if multiple SNPs were assigned to the same chromosomal location in separate datasets. In addition, SNPs with conflicting alleles across datasets were eliminated. For each SNP, a Z-score for association was calculated by β/SE(β) or LN(OR)/SE(LN(OR)), where β, OR, and SE are the beta coefficient, odds ratio, and standard error of the effect estimate, respectively. After variant extraction, the numbers of remaining variants were 23,352,867 for T2D.

### 2.6. Genetic Overlap between Migraine and Headache with T2D

SNP effect concordance analysis (SECA) [26] of GWAS summary statistics was used as our first test for genetic overlap between migraine and T2D and headache and T2D. We used SECA to calculate empirical *p*-values for concordance, defined as an increased agreement in the SNP effects across a pair of GWAS traits. SECA first aligned the SNP effects across the two GWAS summary statistic datasets (dataset 1 and dataset 2) to the same effect allele and extracted a subset of independent SNPs (*r*^2^ < 0.1) using ‘*p*-value informed’ linkage disequilibrium (LD) clumping. The LD-independent SNPs were then partitioned into 12 subsets based on their association *p*-values, where *p* ≤ {0.01, 0.05, 0.1, 0.2, 0.3. 0.4, 0.5, 0.6, 0.7, 0.8, 0.9, 1.0}, yielding 144 subsets from all possible combinations of SNPs for each dataset. Fisher exact tests were carried out on the 144 SNP subsets to assess whether there was an excess of SNPs where the effect directions were concordant across two datasets. More details of the SECA approach are provided in the original publication [26].

We first used migraine and headache GWAS (combined and sex-stratified) as dataset 1 and T2D GWAS (combined and sex-stratified) as dataset 2. We also reversed the datasets using T2D GWAS as dataset 1 and migraine and headache GWAS as dataset 2 to allow for power differences of each GWAS to produce robust *p*-values on which to perform LD-clumping and SNP subsetting.

### 2.7. SNP-Based Heritability and Genome-Wide Genetic Correlation

We applied univariate LD-score regression (LDSC) (https://github.com/bulik/ldsc (accessed on 12 October 2019)) [27] to calculate the proportion of phenotypic variance explained by common genetic variants, a measure called SNP-based heritability, *h*^2^_SNP_. We utilised pre-computed LD scores derived from 1000 Genomes Project European reference genotype data provided by the LDSC developers. Genetic correlations (*r*_g_) were also estimated for combined and sex-specific studies using bivariate LDSC between migraine and headache with T2D. LDSC provides an unbiased estimate of *r_g_*, which may exceed the range (−1 to 1) when standard errors are large and genetic correlations between studies are highly significant. The SNP-based heritability estimates were transformed to the liability scale using the trait sample and population prevalence. Population prevalences of 16% for migraine (18.9% for women, 9.8% for men) [28] and 10% for T2D (9% for women, 9.6% for men) [29] were used. For headache, the observed sample prevalence was used as the population prevalence. There was no significant sample overlap between the migraine, headache, and T2D GWAS; thus, we constrained the genetic covariance intercepts to zero to estimate genetic correlations.

### 2.8. Regional Pleiotropic Genetic Effects

We applied pairwise GWAS (GWAS-PW) to find genomic regions associated with a pair of traits. This program uses a Bayesian statistical model to assess the probability (posterior probabilities of association (PPA)) for four models: (1) the region contains a genetic variant only associated with trait 1 (PPA1); (2) the region contains a genetic variant only associated with trait 2 (PPA2); (3) the region contains a genetic variant that is associated with both traits (PPA3), and (4) the region contains different genetic variants that are separately associated with each trait (PPA4). The posterior probability of each independent genomic region for each pair of traits was calculated using the software GWAS-PW v0.21 [30]. The GWAS summary statistics data were analysed by partitioning them into predefined independent genetic regions (1703 in total) based on LD patterns in the 1000 Genomes Project European reference data provided by the GWAS-PW developers. We considered genomic regions with PPA3 > 0.9 to influence both traits significantly, whereas segments with PPA3 > 0.5 were deemed suggestive.

### 2.9. Cross-Trait Meta-Analysis between Migraine and Headache with T2D

Methods for cross-trait meta-analysis are advantageous for identifying novel genetic variants or loci associated with multiple traits. We performed cross-trait GWAS meta-analyses using two models: the fixed effect (FE) and the modified random effects (RE2) models [31], which are integrated into the METASOFT software (http://genetics.cs.ucla.edu/meta/ (accessed on 10 December 2020)). The FE model used the inverse variance weighted approach to estimate the SNP meta-analysis *p*-value, assuming the GWAS traits examined the same (fixed) effect. Therefore, when SNP effects are heterogeneous, the modified random effects (RE2) model may be more appropriate to estimate the SNP meta-analysis *p*-value. The RE2 model [31] accounts for differences in SNP effects and is robust in the presence of heterogeneity. After a meta-analysis, SNPs and loci that became significant (*P*_meta_ < 5 × 10^−8^) but were not genome-wide significant in any of the two individual trait GWAS datasets (5 × 10^−8^ < *P*_single-trait_ < 0.05) were considered novel. We further evaluated SNPs and loci from the meta-analysis of migraine and T2D, and headache and T2D, using the METASOFT m-value approach [32]. The m-value is the posterior probability that the effect exists in each trait of the cross-trait meta-analysis [32]. M-values greater than 0.9 indicate the effect is present, m-values below 0.1 indicate the effect is not present, and m-values between 0.1 and 0.9 indicate it is ambiguous whether the effect is present.

### 2.10. Identification of Independent Novel Lead SNP or Genomic Loci

FUMA was used to identify independent lead SNPs from a cross-trait meta-analysis of migraine and T2D, and headache and T2D. FUMA is a web-based tool for functional mapping of genetic variants (http://fuma.ctglab.nl/ (accessed on 14 November 2021)) [33]. First, we identified genome-wide significant LD-independent SNPs based on their cross-trait meta-analysis *p*-value (*p* < 5 × 10^−8^) and independence from one another (*r*^2^ < 0.6) within a one Mb window. Next, lead SNPs were classified as significant independent SNPs independent from one another at *r^2^* < 0.1 within a one Mb window. The 1000 Genomes Project European reference panel was used to calculate all LD information [34]. All genome-wide significant lead SNPs in the original GWAS for each trait were considered a known lead variant. Lead SNPs from our cross-trait meta-analyses that were in LD (*r*^2^ > 0.1) with an original GWAS trait SNP were deemed to be within a known locus and excluded. The genome-wide significant lead SNPs from our cross-trait meta-analyses that remained were designated novel lead SNPs. Novel lead SNPs were also examined using PhenoScanner (v2) [35] to see if they or their LD proxies (*r*^2^ > 0.5) were genome-wide significant in published GWAS studies for migraine, headache, or T2D. Finally, we report only novel lead SNPs from cross-trait meta-analyses that were also statistically significant in the original individual trait GWAS at a false discovery rate (FDR) adjusted *p* < 0.05.

### 2.11. Gene-Based Association Analysis to Examine the Genetic Overlap

#### 2.11.1. Gene-Based Test

Compared with single SNP-based association analysis, advantages of gene-based analysis include multiple SNP effects being combined into a single test for a gene and a decreased multiple test burden [36]. Combined and sex-stratified gene-based association tests were carried out between migraine and headache with T2D using the GATES (Gene-Based Association Test Using Extended Simes Procedure) test [37], within the Fast ASsociation Tests (FAST) program [38]. Gene-based *p* values for migraine and T2D, and headache and T2D, were calculated using the SNPs overlapping the migraine and T2D GWAS, and the SNPs overlapping the headache and T2D GWAS, in the combined and sex-stratified dataset. Gene boundaries and positions were taken from the NCBI build 37 (also known as human genome version 19), and the 1000 Genomes Project European phase 3 release was used to estimate LD. SNPs were mapped to 19,418 genes based on the NCBI 37 gene coordinate information, and SNPs found within 10 kb of each gene boundary were allocated to that gene. The gene-based test takes into account the non-independence (LD) between SNPs to adjust for testing multiple SNPs. The GATES test is advantageous because it requires only GWAS summary statistics and a suitable LD reference. It effectively controls the type 1 error rate regardless of gene size and LD pattern among SNPs, and it does not require permutation or simulation to assess empirical significance. The minSNP gene-based test was used, which adjusts the smallest *p*-value associated with the gene by the effective number of independent SNPs assigned to the gene.

#### 2.11.2. Independent Gene-Based Test

Due to LD between the most significant SNP (‘best SNP’) assigned to each gene, gene-based association results may be correlated across neighbouring genes; therefore, the genetic type I error calculator (GEC) [39] was used to estimate the effective number of independent genes (i.e., number of independent gene-based tests) by examining the LD between the top most significant SNP assigned to each gene, and produce the correct type 1 error rate. GEC software was used to perform this analysis as implemented in earlier studies [36,40]. We used the ‘best-SNPs’ identified in our gene-based analyses as GEC input. The GEC method divides the input SNPs into LD blocks, assuming that these blocks are independent by ensuring that the SNPs between the blocks are not in LD (*r*^2^ < 0.1). Analysis of gene-based associations using GEC controls type 1 errors, by correcting for multiple testing using an estimate of the effective number of independent genes [39]. We calculated the number of independent genes for each GWAS trait separately.

#### 2.11.3. Test for Gene-Level Genetic Overlap

We evaluated whether the proportion of associated genes overlapping migraine and T2D, and headache and T2D (combined and sex-stratified) was more than expected by chance at three different nominal *p*-values thresholds (gene with *P*_gene_ ≤ 0.01, *P*_gene_ ≤ 0.05, and *P*_gene_ ≤ 0.1). First, the raw number of overlapping genes was defined as the number of genes overlapping both traits in each of the three *p*-value levels. To assess whether the proportions of overlapping genes were more than expected by chance, we estimated the effective number of independent overlapping genes [36,40,41]. We assigned the migraine or headache GWAS as the ‘discovery’ dataset and the T2D GWAS as the ‘target’ dataset. Then, we conducted independent gene-based tests using only the genes that overlapped both migraine and T2D, and headache and T2D, at each of the three nominal *p*-value thresholds for GEC analysis. The observed number of overlapping genes was defined as the number of effective genes with *p*-values smaller than the threshold in the discovery and target datasets [36,40]. The observed proportion of genes overlapping the pair of traits was calculated by dividing the observed effective number of overlapping genes by the observed effective number of genes in the discovery dataset with a *p*-value less than the threshold [36]. The effective number of genes with *p*-values below the threshold in the target dataset was divided by the total effective number of genes in that dataset to calculate the expected proportion of genes overlapping the two traits [36]. Finally, we used an exact binomial test to compare the proportion of observed and expected overlapping independent genes for the three *p*-value thresholds to determine statistical significance. We examined whether the proportion of overlapping genes was larger than expected by chance [36]. Lastly, we performed a cross-trait gene-based association meta-analysis to identify novel genes associated with migraine and T2D, and headache and T2D. We used Fisher’s combined *p*-value (FCP) approach to combine gene-based association *p*-values across two traits. Several studies recently used this gene-based approach to demonstrate gene-based pleiotropy across different traits [36,40,42,43].

### 2.12. Testing Causal Association

#### 2.12.1. Mendelian Randomisation

We investigated bidirectional causal relationships between migraine and headache with T2D in both combined and sex-stratified datasets using two-sample Mendelian randomisation (2SMR) [44] and generalised summary data-based Mendelian randomisation (GSMR) [45] analyses. For 2SMR analysis, LD-independent (*r*^2^ < 0.001) SNPs with GWAS *p* < 5 × 10^−8^ were used as instrumental variables (IVs). To estimate causal effects, we employed the inverse-variance weighted (IVW) approach as the primary analysis. This method can yield a reliable estimate when horizontal pleiotropy is absent or balanced. However, the IVW model has limitations as one invalid IV can cause the total estimate to be biased. To address this limitation, several complementary MR approaches were used in sensitivity analyses, including MR-Egger [46], MR-weighted median [47], and MR-pleiotropy residual sum and outlier (MR-PRESSO) [48], which have all been reported to be robust to potential violations of IV assumptions. The MR-PRESSO [48] framework was used to conduct a global pleiotropy test. This approach can examine horizontal pleiotropic outliers and determine the adjusted causal effect once these outliers are removed. MR-PRESSO is based on the residual sum of squares (RSS), which measures the heterogeneity of ratio estimates. Specifically, an IVW estimate is computed using the IVs in a leave-one-out manner; if the RSS is considerably different from a simulated Gaussian distribution of predicted RSS, the variant is removed from the IVW model. Simulations showed that this methodology works best when fewer than half of the IVs display horizontal pleiotropy.

We employed GSMR [45] to determine the putative causal direction between migraine and headache with T2D. This method uses summary data to assess the possible causal relationships between the risk factor (exposure) and an outcome utilising independent genome-wide significant SNPs as IVs. The default threshold of GWAS *p* ≤ 5 × 10^−8^ and LD *r*^2^ < 0.05 was used to select independent IVs. The GSMR guidelines recommend using at least ten independent leading SNPs as genetic tools to provide robust results. Therefore, a threshold of *p* ≤ 1 × 10^−5^ was used when less than 10 SNPs exceeded the default threshold. In addition, HEIDI (heterogeneity in the dependent instrument) outlier detection was utilised to filter instruments showing clear pleiotropic impacts on exposure and outcome traits [45]. For the outlier detection analysis in HEIDI, we elected a *p*-value threshold of 0.01, which excludes 1% of SNPs by chance if no pleiotropic outlier effects are found. The method estimates a putative causal effect of the exposure on the outcome (b_xy_) as a function of the relationship between the SNP’s effects on the exposure (b_zx_) and the SNP’s effects on the outcome (b_zy_), given the assumption that the effect of non-pleiotropic SNPs on an exposure (x) should be related to their effect on the outcome (y) in an independent sample only via mediation through the phenotypic causal pathway (b_xy_) [45]. The calculated causal effect coefficients (b_xy_) for the case–control trait are nearly equal to the natural log odds ratio (OR). An odds ratio of 2 is a doubling risk compared to the population prevalence of a binary trait for every standard deviation increase in the exposure trait. This approach can help distinguish the possible causal association between two traits but cannot explain the intermediary mechanisms involved in any possible causation process. This study used the ‘TwoSampleMR’ v0.5.6, ‘MR-PRESSO’ v1.0 packages in R version 4.0.2, and the GSMR analysis, integrated into the GCTA v1.93.2 software [49] to perform all MR analyses. Multiple MR techniques were used in this study due to their different assumptions, strengths, and limitations. Findings supported by multiple MR approaches are considered more robust.

#### 2.12.2. Latent Causal Variable Model

A latent causal variable model (LCV) was also applied to examine whether an observed genetic correlation reflects a causal association. The genetic causality proportion of T2D on risk for migraine and headache was estimated using LCV (https://github.com/lukejoconnor/LCV (accessed on 17 March 2021)) [50]. Although both the GSMR and the LCV models estimate causal inference based on GWAS summary statistics, the GSMR model measures the influence of exposure on the outcome by utilising SNPs strongly associated with exposure. In contrast, the LCV model predicts the genetic causality proportion (GCP) by considering genome-wide SNPs and is robust to horizontal pleiotropy and sample overlap. In essence, the LCV approach implies that a latent variable *L* mediates the genetic correlation between two traits and tests whether this latent variable has a more significant association with one trait than the other [50]. The GCP estimate determines how much genetic causality exists between the two traits. GCP values vary from −1 to 1 (complete genetic causality of trait 2 on trait 1 and vice versa for positive values), with a GCP of zero indicating the detection of horizontal pleiotropy rather than genetic causality. As recommended by the LCV developers, only SNPs with a minor allele frequency (MAF) > 0.05 were retained in the GWAS summary data, and the MHC region was excluded because of its complex LD structure. Before LCV analysis, migraine, headache, and T2D GWAS summary statistics were ‘munged’ to extract only HapMap3 SNPs outside the MHC region (MAF > 0.05). LD scores for HapMap3 SNPs (MHC excluded) estimated from 1000 Genomes Project phase 3 data were used for our LCV analyses.

### 2.13. Pathway Enrichment Analysis of Cross-Trait-Associated Genes

To help characterise the genes identified from cross-trait gene-based association analysis, we used the g:GOst webtool [51,52] from g-profiler to test for enrichment of shared genes in the Gene Ontology (GO) biological process, Kyoto Encyclopedia of Genes and Genomes (KEGG), Reactome, and Wiki-Pathways. The genes overlapping migraine and T2D, and headache and T2D, at *P_gene_* < 0.01 for the combined GWAS and *P_gene_* < 0.1 for the sex-stratified GWAS were used as input to the ‘g:GOSt’ tool to identify pathways with an adjusted enrichment *p*-value (*P*_adj_) < 0.05 [52]. The default and recommended ‘g:SCS algorithm’ was used to determine the *P*_adj_, which controls for multiple testing. In addition, the term sizes of the functional category were limited to values between 5 and 350 [52]. Finally, all advanced options were left as their default values in our analyses.

## 3. Results

### 3.1. Genetic Overlap of Migraine and Headache with T2D

**Figure 2:**
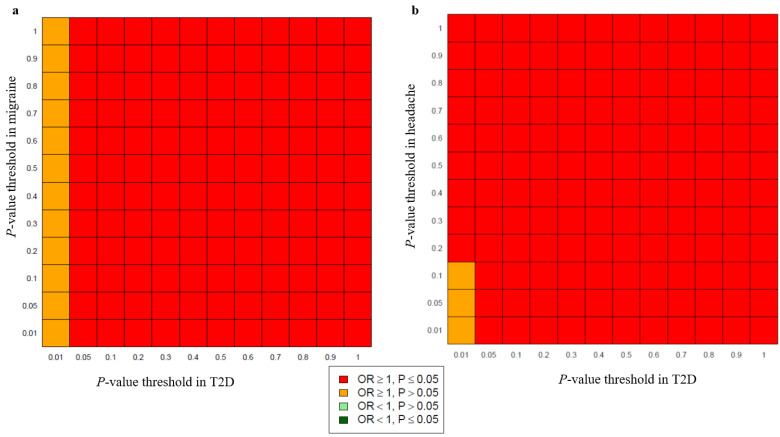
Genetic overlap between migraine and T2D, and headache and T2D. Heatmap plots from SECA exact Fisher tests for SNP effect concordance between (**a**) migraine and T2D, and (**b**) headache and T2D. Among the 144 SNP subsets examined, 132 and 141 SNP subsets (red colour in the figure) showed significant concordant effects (OR > 1 and *P* < 0.05) between migraine and T2D and between headache and T2D, respectively.

Analysis of the combined and sex-specific GWAS data using SECA produced evidence for significant genetic overlap (*p* = 9.99 × 10^−4^) between migraine and T2D, and between headache and T2D, with an excess of SNPs where the effect directions are concordant, indicating that SNPs associated with an increase in migraine and headache risk are also associated with an increased risk of T2D (Figure 2). Of the 144 SNP subsets examined, 132 demonstrated nominally significant concordant effects (OR > 1 and *p* < 0.05) across migraine (dataset 1) and T2D (dataset 2), while 141 SNP subsets showed significant concordant effects across headache (dataset 1) and T2D (dataset 2). SECA provided results that followed a similar pattern in reverse analyses using T2D GWAS data as dataset 1 and headache or migraine GWAS data as dataset 2. SNPs with lower (more significant) association *p*-values for migraine and headache exhibited a statistically significant trend towards lower *p*-values in T2D. In line with expectations for true genetic overlap, an increased concordance was observed in SNP subsets with more robust effect estimates (i.e., represented by more significant GWAS *p*-values) (Supplementary Table S2). In addition, we observed a significant genetic overlap between headache and T2D in males (*p* = 9.99 × 10^−4^). Our SECA results revealed an effect concordance across headache (dataset 1) and T2D (dataset 2) in males, with 102 SNP subsets displaying nominally significant concordant effects. Analogous results were obtained using the male T2D GWAS data as dataset 1 and the headache GWAS data as dataset 2. In contrast, no genetic overlap was detected between headache and T2D in females, nor between migraine and T2D in males and females. A summary of all SECA analyses is presented in supplementary data (Supplementary Table S2).

### 3.2. Genetic Correlations of T2D with Migraine and Headache

Single-trait LDSC estimated a SNP-based heritability on a liability-scale (*h*^2^_SNP_) (without constrained intercept) of 11.08% (95% CI: 10.24–11.92%) for migraine, 9.03% (95% CI: 8.23–9.83%) for headache, and 17.23% (95% CI: 15.72–18.74%) for T2D. Sex-specific analyses produced similar heritability estimates for males (IHGC-migraine: *h*^2^_SNP_ = 11.25% [95% CI: 2.16–20.34%]; UKB-headache: *h*^2^_SNP_ = 8.57% [95% CI: 7.39–9.75%]; UKB-T2D: *h*^2^_SNP_ = 25.33% [95% CI: 21.57–29.09%]) and females (IHGC-migraine: *h*^2^_SNP_ = 10.1% [95% CI: 7.63–12.57%]; UKB-headache: *h*^2^_SNP_ = 10.01% [95% CI: 8.95–11.07%]; UKB-T2D: *h*^2^_SNP_ = 24.15% [95% CI: 19.00–28.31%]). Given the observed significant evidence for SNP-based heritability, we tested for genetic correlation between migraine and headache with T2D (combined and sex-stratified) using bivariate LDSC. Bivariate LDSC analyses of the combined GWAS data estimated a significant genetic correlation between T2D and migraine (*r*_g_ = 0.06; *p* = 1.37 × 10^−5^), and T2D and headache (*r*_g_ = 0.07; *p* = 3.0 × 10^−4^). Analogous to the SECA results, we also identified a significant genetic correlation between T2D and headache in males (*r_g_* = 0.09; *p* = 9.4 × 10^−3^) but not in females and found no evidence for a genetic correlation between migraine and T2D in males and females. The SNP-based genetic correlation and heritability estimates for migraine and headache with T2D are summarised in Table 1 and Supplementary Table S3, respectively.

### 3.3. Pairwise GWAS of Migraine and Headache with T2D

GWAS-PW analysis revealed 11 significant pleiotropic regions for migraine and T2D (chr1:177434054–178944161, chr3:8648561–9541905, chr4:2844097–3845571, chr5:73759326–75795407, chr6:160581374–162169452, chr9:135298917–137040737, chr11:47008125–49865926, chr14:57482514–59448252, chr14:94325812–95750857, chr15:53069747–54508497, and chr17:59312894–61545486) having PPA3 > 0.9, indicating that these regions are pleiotropic and likely harbour a single causal variant influencing both traits. For headache and T2D, significant pleiotropy (PPA3 > 0.9) was identified in five regions (chr1:153181186–154770139, chr3:70449145–72528844, chr5:73759326–75795407, chr7:1353067–2062006, and chr14:94325596–95750857). Notably, two of these pleiotropic regions (chr5:73759326–75795407 and chr14:94325596–95750857) were common to both migraine and T2D, and headache and T2D. Additionally, we identified 35 and 13 pleiotropic genomic regions between migraine and T2D, and between headache and T2D at PPA3 > 0.5; seven regions were common to both (Supplementary Table S4). These findings provide further support for shared genetic factors across migraine, headache, and T2D and highlight specific genomic regions harbouring causal genetic variants influencing both migraine and T2D, both headache and T2D, and all three traits. Table 2 summarises the combined GWAS-PW results for migraine and headache with T2D. There were no significant (PPA3 > 0.9) pleiotropic regions in the sex-stratified GWAS-PW analysis. Relaxing the threshold to PPA3 > 0.5 found one pleiotropic region between migraine and T2D in females, but none in males (Supplementary Table S4). Whereas for headache and T2D, three pleiotropic regions were found in females and only one region was found in males (Supplementary Table S4). Interestingly, the one pleiotropic region for headache and T2D found in males (chr6:28018353–28917091) was one of the three regions found in females. The differences in the pleiotropic genomic regions between migraine and headache with T2D in males and females may be related to their different GWAS sample sizes.

### 3.4. Identification of Novel Lead SNPs between Migraine and Headache with T2D

Following the exclusion of genome-wide significant SNPs in the corresponding original single-trait GWAS, and SNPs in LD (*r*^2^ ≥ 0.1) with these SNPs, our cross-trait GWAS meta-analysis identified 23 LD-independent novel lead SNPs for migraine and T2D. These lead SNPs achieved genome-wide significance in the cross-trait meta-analysis (*P*_meta_ < 5 × 10^−8^) and nominal significance (FDR-adjusted *p* < 0.05) in the original single trait GWAS (Table 3). The posterior probability (m-value) results indicated that all detected lead SNPs were associated with both migraine and T2D (i.e., the m-value was equal to one for both migraine and T2D). The top significant SNP was rs11590235 (*P*_meta_ ≤ 3.11 × 10^−12^), located at the *DENND1A* locus. Cross-trait GWAS meta-analysis identified three novel lead SNPs significantly associated with headache and T2D (Table 4). The posterior probability (m-value = 1) results indicate that the identified lead SNPs are associated with both headache and T2D. The most significant locus for headache and T2D was near *ELFN1* (lead SNP: rs73050128, *P*_meta_ ≤ 1.50 × 10^−11^). Notably, two of these lead SNPs, rs546738 and rs73050128, mapped to regions near the protein-coding genes *NLGN1* and *ELFN1*, respectively. These two gene regions were also implicated in the cross-trait GWAS meta-analysis of migraine and T2D, by rs536445 and rs62442924, respectively; and SNPs rs546738 and rs536445, and SNPs rs73050128 and rs62442924, were in significant LD (*r*^2^ = 0.87 and *r*^2^ = 0.76, respectively). Results for all novel lead SNPs found in the cross-trait meta-analysis between migraine and T2D (38 lead SNPs), and headache and T2D (17 lead SNPs), and an unadjusted *p* < 0.05 in their respective single-trait GWAS, are provided in Supplementary Tables S5 and S6.

### 3.5. Utility of the Cross-Trait GWAS Meta-Analysis Approach

To assess the utility of cross-trait GWAS meta-analysis to identify novel risk loci, we performed a cross-trait GWAS meta-analysis of migraine and T2D, using a previous and less powerful migraine GWAS dataset by Gormley et al. (2016) [3]. Of the 25 novel lead SNPs (*P*_meta_ < 5 × 10^−8^, single-trait *p* < 0.05) identified from cross-trait meta-analysis of the 2016 migraine GWAS [3] and T2D GWAS [17], 15 (60%) had a more significant *p*-value in the recent and more powerful Hautakangas et al. (2022) migraine GWAS [2] than in the Gormley et al. (2016) migraine GWAS, of which four SNPs (rs2150866, rs169381, rs9894634, and rs2834435) became genome-wide significant, and an additional three SNPs (rs11140324, rs2670139, and rs11646063) became genome-wide suggestive (*p* < 1 × 10^−5^) (Supplementary Table S7). Furthermore, of the six SNPs (*P*_meta_ < 5 × 10^−8^, single-trait FDR-adjusted *p* < 0.05) identified from cross-trait meta-analysis, five (83%) had a more significant *p*-value in the recent and more powerful migraine GWAS [2], of which two (33%) SNPs (rs2150866 and rs169381) became genome-wide significant, and an additional SNP (rs11140324) became genome-wide suggestive (*p* < 1 × 10^−5^) (Supplementary Table S7). These results provide important proof-of-principle that our cross-trait GWAS meta-analysis approach identifies true novel risk loci.

### 3.6. Gene-Based Genetic Overlap between Migraine and Headache with T2D

The results presented in Table 5 and Table 6, respectively, describe a significant genetic overlap between migraine and T2D, and between headache and T2D at the gene level. GATES gene-based association analysis of the migraine and T2D GWAS data produced results for 18,309 genes, while results for 18,261 genes were produced for headache and T2D analysis. Our results demonstrated a significant gene-level genetic overlap between migraine and headache with T2D across all three *p*-value thresholds using a binomial test for an increase in genes associated across the traits (Table 5 and Table 6). For example, the observed proportion (0.403) of genes with a gene-based *p* value ≤ 0.05 for both migraine and T2D was significantly larger than the expected proportion (0.281) (*P*_binomial-test_ = 2.83 × 10^−46^). Similarly, the observed proportion (0.412) of genes with a gene-based *p* value ≤ 0.05 for both headache and T2D was significantly larger than the expected proportion (0.287) (*P*_binomial-test_ = 4.08 × 10^−29^). Significant gene-based genetic overlap was also observed for both the male- and female-specific analysis of headache and T2D, and the female-specific analysis of migraine and T2D (Supplementary Table S8).

For the migraine and T2D overlap analysis, we used a genome-wide significant threshold of *P_gene_* < 3.63 × 10^−6^ for migraine (i.e., Bonferroni adjustment for testing 13,757 effectively independent gene-based association tests [0.05/13,757]) and *P*_gene_ < 3.65 × 10^−6^ for T2D (i.e., Bonferroni adjustment for testing 13,694 effectively independent genes [0.05/13,694]) and identified 303 genome-wide significant genes associated with migraine (Supplementary Table S9a) and 607 genes associated with T2D. For the headache and T2D overlap analysis, we used a genome-wide significant threshold of *P_gene_* < 3.85 × 10^−6^ for headache (Bonferroni adjustment for testing 13,002 effectively independent genes [0.05/13,002]) and *P_gene_* < 3.65 × 10^−6^ for T2D (Bonferroni adjustment for testing 13,694 effectively independent genes [0.05/13,694]) and identified 161 genes associated with headache (Supplementary Table S9b) and 592 genes associated with T2D. At the genome-wide significant level, a total of 33 genes were associated with both migraine and T2D (Table 7), and ten genes were associated with both headache and T2D (Table 8). Notably, seven of these genes (*EHMT2*, *SLC44A4*, *PLEKHA1*, *CFDP1*, *TMEM170A*, *CHST6*, and *BCAR1*) were genome-wide significant for all three traits (migraine, headache, and T2D). Using the FCP approach, we combined evidence for a gene-based association for genes overlapping migraine and T2D, and headache and T2D, with an FDR-adjusted single-trait *P*_gene_ < 0.1. FCP results show that 440 genes overlapping migraine and T2D (*P*_Fisher’s-combined_ < 3.63 × 10^−6^), and 204 genes overlapping headache and T2D (*P*_Fisher’s-combined_ < 3.65 × 10^−6^) reached a gene-based genome-wide significant level (Supplementary Tables S10 and S11). Of the 440 genes overlapping migraine and T2D, 165 had their top SNP associated with both traits at a genome-wide suggestive significant level (*P_SNP_* < 1 × 10^−5^). Of the 204 genes overlapping headache and T2D, 78 had their top SNP associated with both traits at a genome-wide suggestive significant level (Supplementary Tables S10 and S11). No genome-wide significant genes overlapped migraine and T2D, or headache and T2D, in the male- and female-specific analyses—most likely due to a lack of power in the sex-specific GWAS datasets. Therefore, we did not perform FCP for the genes overlapping migraine and T2D, and headache and T2D.

### 3.7. Causal Inference between Migraine and Headache with T2D

**Figure 3:**
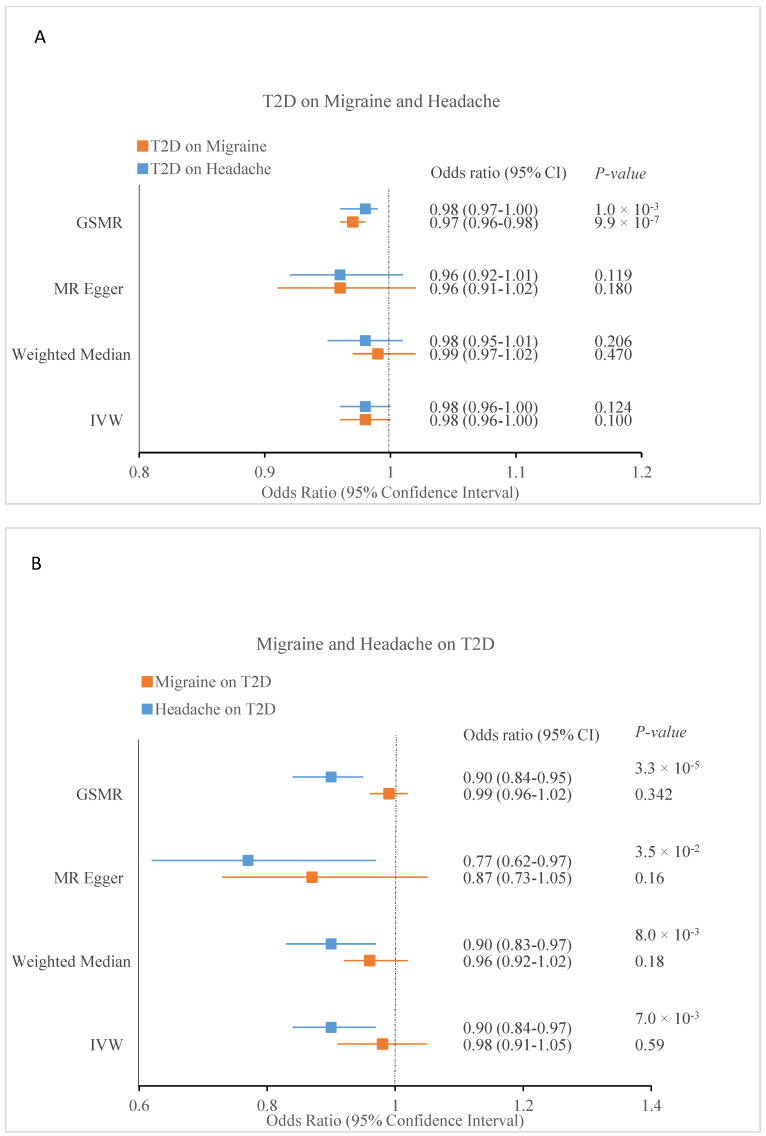
The forest plot presents ORs and 95% CIs for MR analyses testing genetic predisposition to T2D on migraine and headache (**A**), and genetic predisposition to migraine and headache on T2D (**B**). IVW: Inverse variance weighted, GSMR: Generalised summary data-based Mendelian Randomisation, T2D: Type 2 Diabetes.

The results of our MR analysis examining the causal relationship between migraine and T2D, and headache and T2D are summarised in Table 9. 2SMR analysis using the IVW MR model found no evidence for a significant causal association (OR = 0.98, 95% CI: 0.96–1.00, *p* = 0.1) between T2D (exposure variable) and migraine (outcome variable). The results of the weighted median (OR = 0.99, 95% CI: 0.97–1.02, *p* = 0.47) and MR-Egger (OR = 0.96, 95% CI: 0.91–1.02, *p* = 0.18) models were similar to the IVW result (Figure 3 and Table 9). There was significant evidence of heterogeneity (Cochran’s Q *P*_ivw_ = 4.59 × 10^−50^); however, the MR-Egger intercept (*p* = 0.48) showed that the observed heterogeneity was not the result of horizontal pleiotropy. The raw estimate from MR-PRESSO after removing eight outliers (OR = 0.98, *p* = 0.11) also agreed with the IVW results, and the MR-PRESSO ‘global test’ showed significant evidence for horizontal pleiotropy (*P*_global-test_ < 2 × 10^−5^). In contrast, GSMR found significant evidence for a causal association (OR = 0.97, 95% CI: 0.96–0.98, *p* = 9.92 × 10^−7^). Reverse 2SMR analyses also found no evidence for a causal association of migraine (exposure variable) on T2D (outcome variable) utilising the IVW (OR = 0.98, 95% CI: 0.91–1.05, *p* = 0.59), weighted median (OR = 0.96, 95% CI: 0.92–1.02, *p* = 0.18), and MR-Egger models (OR = 0.87, 95% CI: 0.73–1.05, *p* = 0.16). MR-PRESSO (OR = 1.00, *P*_global-test_ < 2 × 10^−5^) and GSMR (OR = 0.99, 95% CI: 0.96–1.02, *p* = 0.34) both support this finding (Figure 3 and Table 9).

2SMR analysis found no evidence to support a causal effect of T2D (exposure variable) on headache (outcome variable) using the IVW (OR = 0.98, 95% CI: 0.96–1.00, *p* = 0.12), weighted median (OR = 0.98, 95% CI: 0.95–1.00, *p* = 0.21), MR-Egger (OR = 0.96, 95% CI: 0.92–1.01, *p* = 0.12), and MR-PRESSO (OR = 0.99, *p* = 0.19) models. While GSMR analysis found some evidence for a causal effect of T2D on headache (OR = 0.98, 95% CI: 0.97–1.00, *p* = 0.01). Reverse 2SMR found some evidence for a causal association of headache on T2D using the primary IVW model (OR = 0.90, 95% CI: 0.84–0.97, *p* = 7 × 10^−3^); moreover, in line with the IVW model, the weighted median (OR = 0.90, 95% CI: 0.83–0.97, *p* = 8 × 10^−3^) and MR-Egger (OR = 0.77, 95% CI: 0.62–0.97, *p* = 3.5 × 10^−2^) sensitivity analyses also found significant evidence for causal association. There was evidence for heterogeneity (Cochran’s Q *P*_ivw_ = 3.21 × 10^−3^), although the MR-Egger intercept (*p* = 0.17) indicated that this was not likely caused by horizontal pleiotropy. Similarly, MR-PRESSO produced a significant raw estimate after excluding two outliers (OR = 0.90, *p* = 7 × 10^−5^) and a global test for horizontal pleiotropy (*P*_global-test_ = 4.7 × 10^−3^). Furthermore, GSMR found significant evidence for a causal association (OR = 0.90, 95% CI: 0.84–0.95, *p* = 3.32 × 10^−5^) (Figure 3 and Table 9).

Using the same MR methods, we tested for sex-specific causal association(s) between migraine and T2D, and headache with T2D (Table 9). In males, we found no significant evidence for a causal effect of T2D on migraine or a causal effect of migraine on T2D. In females, only GSMR analysis found evidence for a causal effect of T2D on migraine (OR = 0.93, *p* = 5.0 × 10^−3^). Reverse MR analyses found no significant evidence for a causal effect of migraine on T2D. In contrast, IVW, MR-PRESSO, and GSMR found significant evidence for a causal effect of T2D on headache in females, and no causal association in males, whereas reverse MR analyses found no significant evidence for a causal effect of headache on T2D in females, and IVW and MR-PRESSO found evidence for a causal effect of headache on T2D in males.

Given the significant genetic correlation between migraine and T2D in the sex-combined LDSC analysis and the significant genetic correlation between headache and T2D in sex-combined and male-specific LDSC analyses, we used the LCV approach to test for a significant genetic causality proportion of T2D on risk for migraine or headache, defined as the mean posterior estimate of the GCP. The GCP estimate for migraine (GCP = 0.27, SE = 0.47, *p* = 0.32) and for headache (GCP = 0.15, SE = 0.49, *p* = 0.74) with T2D indicates that T2D is not genetically causal for migraine and headache, and vice versa (i.e., the sign of the GCP parameter indicates causal direction). Similarly, LCV found no evidence (GCP = −0.08, SE = 0.52, *p* = 0.98) for genetic causality between T2D with headache in males (Supplementary Table S12). The genetic instruments used for migraine, headache, and T2D in the sex-combined 2SMR and GSMR analyses are also provided in Supplementary Tables S13–S16.

### 3.8. Pathway Enrichment Analysis of Genes Associated across Migraine and Headache with T2D

Pathway-based analysis was performed to identify biological pathways enriched for genes overlapping migraine and T2D (662 genes), and headache and T2D (353 genes) with a *P_gene_* < 0.01 in the sex-combined analyses. For the sex-stratified pathway analyses, we used genes overlapping migraine and T2D, and headache and T2D with a *P_gene_* < 0.1. For the migraine and T2D, and headache and T2D overlapping genes, 90 and 118 biological pathways or processes were significantly enriched, respectively. Pathways related to cell signalling (e.g., ‘signalling by notch’), cellular processes, epigenetic mechanisms, oxidative stress, and immune system (e.g., ‘systemic lupus erythematosus’) were observed to be significantly (*P*_(adjusted)_ < 0.05) enriched for both migraine and T2D, and headache and T2D overlapping genes (Supplementary Tables S17 and S18). Additional information, including the genes involved in these pathways, can be found in Supplementary Tables S17 and S18. Pathway enrichment analyses for headache and T2D identified 26 and 161 biological pathways that were significantly (*P*_(adjusted)_ < 0.05) enriched in females (Supplementary Table S19) and males (Supplementary Table S20), respectively. No pathway was significantly enriched by the genes overlapping migraine and T2D in both males and females.

## 4. Discussion

This deep investigation using large and well-powered GWAS datasets provides new and important insight into the genetic relationship between migraine, headache, and T2D. We observed similar significant genetic correlations between migraine and T2D (*r*_g_ = 0.0589; 95% CI: 0.0324–0.0854) and headache and T2D (*r*_g_ = 0.0657; 95% CI: 0.03–0.1014), indicating shared genetic factors contribute to the co-occurrence of migraine and headache with T2D. These findings substantiate previous observational studies that found a higher co-occurrence of T2D with migraine [8] and headache [53]. Additionally, these findings confirm and extend the recent report of a positive genetic correlation between T2D and migraine (*r*_g_ = 0.09, *p* = 0.004) [12], using independent GWAS datasets. Interestingly, our sex-stratified analyses identified a significant genetic correlation between headache and T2D in males (*r_g_* = 0.0922; 95% CI: 0.0226–0.1618) but not in females (*r_g_* = 0.0049; 95% CI: −0.0727–0.0825), and found no evidence for a genetic correlation between migraine and T2D in males or females. Considering the increased prevalence of migraine and headache in females compared to males [54,55], and larger female migraine and headache GWAS sample sizes, this finding suggests that the relationship between migraine, headache, and T2D is stronger in males, and the relationship between migraine and T2D is perhaps driven by the symptom of headache pain rather than the other symptoms of migraine.

GWAS-PW analysis identified several specific genomic regions shared between migraine and headache with T2D (Table 2). A total of 11 pleiotropic regions were found across migraine and T2D. While five pleiotropic regions were found across headache and T2D, of which two were among the 11 pleiotropic regions associated with migraine and T2D. These two common regions harbour genetic variants mapped to several gene-based genome-wide significant genes with top SNPs *p* < 1.0 × 10^−5^ (*ANKDD1B*, *POC5*, *SERPINA1*, *HMGCR*, and *COL4A3BP*) (Table 2)*. ANKDD1B* and *SERPINA1* were the nearest genes to a genome-wide significant migraine risk SNP [2], and *POC5* was the nearest gene to a genome-wide significant T2D risk SNP [17]. Identifying these pleiotropic gene associations provides leads on the likely underlying biological mechanisms and thus improves our understanding of their role in migraine and T2D. More generally, pleiotropy has applications in drug discovery and genomic editing [56]. For example, *ANKDD1B*, a T2D-related gene, is involved in metabolism and inflammation [57], which may play a role in the pathophysiology of migraine, as previous research reported that inflammatory processes are associated with migraine [58]. No significant pleiotropic regions (PPA3 > 0.9) were found across migraine and T2D, and headache and T2D, in the male- and female-specific GWAS datasets (Supplementary Table S4). However, this may be due to the reduced power in the sex-specific GWAS datasets to identify genome-wide significant SNP loci; indeed, relaxing the pleiotropy threshold to PPA3 > 0.5 found suggestive evidence for pleiotropic association. Therefore, GWAS-PW analysis of larger female- and male-specific GWAS datasets will be required to identify significant (PPA3 > 0.9) sex-specific pleiotropic regions are shared between migraine and headache with T2D.

We used genome-wide cross-trait meta-analysis to enhance our power to identify novel SNPs significantly (*p* < 5 × 10^−8^) associated with migraine, headache, and T2D. We found 23 novel lead SNPs associated with migraine and T2D, and three novel lead SNPs associated with headache and T2D, that were not at genome-wide significant loci in the respective single-trait GWAS. Notably, two of these novel lead SNP loci, near the protein-coding genes *NLGN1* and *ELFN1*, overlapped. The *NLGN1* (neuroligin 1) gene is abundant at excitatory synapses and is essential for synaptic function. In addition, *NLGN1* recently revealed high expression in vascular endothelial cells and was reported to play a crucial function in vascular development [59]. Therefore, the downregulation of *NLGN1* in endothelial cells may thus play a significant role in the pathogenesis of endothelial cell dysfunction in T2D. Furthermore, clinical investigations have shown that genetic variants of *NLGN1* are associated with neuropsychiatric conditions such as autism spectrum disorder, memory loss and depression in Alzheimer’s disease, and post-traumatic stress disorder, indicating a role for *NLGN1* in multiple neurological disorders [60]. *ELFN1* (extracellular-leucine-rich repeat fibronectin domain 1) is highly expressed in GABAergic (γ-aminobutyric acid-ergic) interneurons in the hippocampus and is involved in the recruitment of metabotropic glutamate receptors, such as mGluR7, to the presynaptic membrane [61]. Mutant mice with *ELFN1* knocked out experience seizures [62]. Overexcitation in one or more brain regions can cause migraine, headache, and seizures. Of the protein-coding genes near the novel lead SNPs from analysis of migraine and T2D (Table 3), *L3MBTL2* and *DENND1A* are also particularly interesting. For example, *L3MBTL2* is a transcriptional repressor expressed in adipose, brain, heart, lung, and muscle; and was very recently implicated in migraine, schizophrenia, and depression via cross-trait genetic analysis [63]. Positional and eQTL mapping revealed differential expression of this gene in the anterior cingulate and frontal cortex [63]. The frontal cortex is the brain region affected in migraine patients [64]. Neurological conditions such as depression are associated with elevated endoplasmic reticulum (ER) stress. Dysregulation of the ER stress response is involved in the pathogenesis of numerous diseases, including T2D and cancer [65]. The protein encoded by *DENND1A* is involved in endosomal membrane trafficking [66]. Recent research has shown that *DENND1A* is associated with insulin resistance (IR) in women with polycystic ovarian syndrome, indicating that metabolic dysfunction may play a pathophysiologic role [67]. Interestingly, migraine is associated with an increased risk of metabolic dysregulation, including IR [11,68]. Therefore, they are plausible biologically candidate genes for migraine and T2D. Furthermore, 13 of the novel lead SNPs identified in the cross-trait meta-analyses were within the pleiotropic genomic regions identified by GWAS-PW (Supplementary Tables S5 and S6).

One genome-wide significant lead SNP (rs10457469, near *HEY2*) from the cross-trait meta-analysis of migraine and T2D was not novel (Supplementary Table S5) as, although it was genome-wide suggestive in the individual migraine GWAS, it was genome-wide significant in the individual T2D GWAS [17]. Furthermore, the *HEY2* gene is significantly expressed in vascular tissues and plays a role in vascular function; and was implicated in a previous migraine [3] and recent Brugada syndrome (a potentially fatal heart rhythm disease) GWAS [69]. PhenoScanner identified two novel lead SNPs associated with migraine and T2D (rs4809370, rs62442924) and one novel lead SNP associated with headache and T2D (rs73050128) are also associated with hypertension, blood pressure, body mass index, and vascular heart problems. In addition, three novel lead SNPs associated with migraine and T2D (rs62442924, rs171697, and rs1841499) were also associated with depression and neuroticism.

Our gene-based analyses identified 33 and 10 genes significantly associated (*P*_gene_ < 3.85 × 10^−6^) with migraine and T2D, and headache and T2D, respectively. Among the overlapping genes between migraine and T2D, seven genes (*MACF1*, *CALCB*, *THADA*, *ANKDD1B*, *EHMT2*, *CFDP1*, and *SUGP1*) were the closest gene to a lead migraine SNP in Hautakangas et al. (2022) [2]. While eight of the 33 shared genes (*MACF1*, *THADA*, *SLC9B1*, *POC5*, *PLEKHA1*, *CELF1*, *BCAR1*, *EYA2*) were the closest gene to a lead T2D SNP in Mahajan et al. (2018) [17]. For example, the *ANKDD1B* gene encodes a protein called ankyrin repeat and death domain-containing 1 B. Recent studies have shown that the *ANKDD1B* gene is involved in vascular and endothelial function and contributes to migraine and blood pressure risk [4]. Although there is little data on *ANKDD1B*, it may be relevant to the pathophysiology of migraine and T2D due to its role in vascular function. Five of the 10 genes overlapping headache and T2D (*SLC44A4*, *EHMT2*, *PLEKHA1*, *CFDP1*, and *TMEM170A*) were previously associated with T2D [17]. Similarly, *PLEKHA1* and *BCAR1* overlapping headache and T2D were previously associated with headache [16]. Hence, variation of the genes identified from our cross-trait analyses may explain, at least in part, the co-occurrence of migraine, headache, and T2D.

Our MR analyses found inconsistent evidence for a causal effect of genetic liability to T2D on migraine and headache, with only GSMR producing significant evidence for T2D having a negative causal (i.e., ‘protective’) effect on both migraine and headache. This inconsistency may be due to the insufficient power of the T2D SNP IVs to detect a causal association using 2SMR. However, the GSMR finding is consistent with previous evidence from observational studies showing that T2D patients are less likely to develop migraine and headaches [70,71,72]; however, the only other known MR study found no evidence of a causal association of T2D with migraine, albeit using smaller sample sizes than utilised here [12]. A plausible explanation for T2D being protective against migraine and headache could relate to the decreased expression of calcitonin gene-related peptide (CGRP) reported in diabetic patients [73]. Several studies demonstrate that CGRP plays an important role in the pathogenesis of migraine via activation of the trigeminovascular system, where increased CGRP levels cause vasodilation and neurogenic inflammation, causing the head pain of a migraine attack [74]. Therefore, decreased CGRP levels in diabetic patients may protect against vasodilation and neurogenic inflammation and hence protect against migraine and headache [70,75,76]. Additionally, lowered nitric oxide and substance *p* levels in diabetic patients were proposed as a potential mechanism for this association [77,78]. Therefore, the possibility that T2D has a protective effect on migraine and headache remains plausible and warrants further analysis, including genetic causality analyses using future more-powerful GWAS datasets.

Reverse MR analyses using 2SMR and GSMR found no evidence for a causal effect of migraine on T2D. In contrast, reverse 2SMR and GSMR analyses produced significant evidence for headache having a negative causal (i.e., ‘protective’) effect on T2D. These findings were surprising given the positive genetic correlation between migraine and T2D, and headache and T2D. A possible explanation for this result could be that MR relies on a subset of genetic variants, whereas genetic correlations quantify the average sharing of genetic effects between two traits across the entire genome. Therefore, MR may detect specific relationships that may be ‘washed out’ by other (including opposing) effects when estimating genome-wide correlation, which may explain why our LCV analyses (which are based on the genome-wide genetic correlation estimate) found no causal relationship between migraine and headache with T2D. Previous observational studies reported that migraine patients have a decreased risk of developing T2D [10]; however, fewer studies are available for headache and T2D. However, given that migraine and headache are genetically highly correlated and likely share similar mechanisms [16], a plausible mechanism for a protective effect of headache on T2D may again relate to CGRP. For example, increased levels of CGRP in the sensory nerve fibres during headache attacks [74], may relate to the aberration of glucose metabolism [10]. Interestingly, recent studies reported that CGRP, and the related peptide amylin, are found in the pancreas, where their function appears to influence insulin secretion from the β-cells and reduce the risk of developing T2D [78]. Lastly, sex-stratified analyses found no consistent evidence of a female- or male-specific causal association between migraine and headache with T2D; however, given their considerably smaller GWAS sample sizes, we encourage sex-specific genetic causality analyses using future more-powerful GWAS datasets to identify sex-specific causal relationships.

Finally, 90 and 118 biological pathways/processes were found to be enriched for genes associated with migraine and T2D, and headache and T2D (Supplementary Tables S17 and S18), respectively. Notably, 86 of the 90 pathways enriched by genes overlapping migraine and T2D, were also enriched by genes overlapping headache and T2D (Supplementary Tables S17 and S18). Thus, indicating similar pathogenetic pathways contribute to the co-occurrence of both migraine and T2D, and headache and T2D.

### Strength and Limitations

Our study has various strengths. This is the first comprehensive study to explore the genetic overlap and causal relationship between migraine and headache with T2D. The analysis utilised the latest and largest (most powerful) available GWAS summary statistic datasets. Second, we analysed people of European descent to minimise the impact of genetic heterogeneity related to ancestry. We have used the latest and largest summary data for migraine in this study, and there are no known replication samples exist. Furthermore, our findings are more robust since they were based on genetic data than prior observational research that may have produced unreliable or unclear results due to small sample numbers, or confounding influences from lifestyles or the environment. 

This study had some limitations. Firstly, the GWAS datasets only included people of European ancestry; hence we cannot determine how relevant these findings are to other non-European populations. However, we were restricted to performing our analyses using the available datasets. Unfortunately, adequate migraine and headache GWAS data are currently not available for population samples of non-European ancestries.

## 5. Conclusions

In conclusion, our findings robustly confirm the comorbidity of migraine and headache with T2D, with shared genetically regulated biological mechanisms driving their co-occurrence, and evidence for a causal relationship between headache and T2D. We identified novel risk SNP and gene loci that provide new biological insight and intervention targets underlying migraine, headache, and T2D. Future functional investigations focusing on specific loci reported through this cross-trait genetic analysis may provide further insights into the biological mechanism underlying the risk of migraine and T2D. Our findings provide important motivation for designing novel treatment strategies to manage T2D in migraine and headache patients.

## Figures and Tables

**Table 1:** Combined and sex-stratified genetic correlation between migraine and headache with T2D on a liability scale.

Trait 1	Trait 2	Rg	SE	*p*
Migraine	T2D	0.0589	0.0135	1.37 × 10^−5^
Migraine (Male)	T2D (Male)	0.0007	0.0759	0.9929
Migraine (Female)	T2D (Female)	0.0743	0.0537	0.1663
Headache	T2D	0.0657	0.0182	3.0 × 10^−4^
Headache (Male)	T2D (Male)	0.0922	0.0355	9.4 × 10^−3^
Headache (Female)	T2D (Female)	0.0049	0.0396	0.9009

T2D: Type 2 diabetes; Rg: genetic correlation (*r*_g_) estimate; SE: standard error of genetic correlation estimate; *p*: *p*-value for test of Rg being different from zero.

**Table 2:** Pleiotropic genomic regions (posterior probability of association [PPA3] > 0.9) of migraine and headache with T2D using GWAS pairwise analysis.

Trait 1	Trait 2	Region	Chr	Start bp	End bp	Locus	PPA1	PPA2	PPA3	PPA4	Genome-Wide Significant Genes Overlapping both Traits in the Highlighted Region (*P*_single trait_ FDR < 0.1 and *P*_meta_ FCP < 3.65 × 10^−6^)
Migraine	T2D	88	chr1	177434054	178944161	1q25.2	0.00	0.00	0.99	0.01	* SEC16B
		283	chr3	8648561	9541905	3p26.1–p25.3	0.00	0.02	0.91	0.07	* SETD5, * LHFPL4
		402	chr4	2844097	3845571	4p16.3	0.00	0.00	1.00	0.00	* ADD1, * MFSD10, * NOP14, * HTT, * MSANTD1
		564 ^a^	chr5	73759326	75795407	5q13.3	0.00	0.00	0.93	0.07	*GCNT4*, *ANKRD31*, * HMGCR, * COL4A3BP, * POLK, * ANKDD1B, * POC5
		734	chr6	160581374	162169452	6q25.3–q26	0.00	0.01	0.95	0.04	* SLC22A3
		1005	chr9	135298917	137040737	9q34.13–q34.20	0.00	0.00	0.97	0.03	* ABO, *BRD3*
		1123	chr11	47008125	49865926	11p11.2–p11.12	0.00	0.00	0.97	0.03	*DDB2*, *MYBPC3*, * SPI1, *SLC39A13*, *PSMC3*, * RAPSN, * CELF1, * PTPMT1, * KBTBD4, * NDUFS3, * FAM180B, * C1QTNF4, * MTCH2, * AGBL2, * FNBP4, * NUP160
		1348	chr14	57482514	59448252	14q22.3–q23.1	0.00	0.00	0.96	0.04	* PSMA3, * ACTR10, * ARID4A, *TOMM20L*, *TIMM9*
		1370 ^a^	chr14	94325812	95750857	14q32.12–q32.13	0.00	0.00	1.00	0.00	*SERPINA2*, * SERPINA1
		1400	chr15	53069747	54508497	15q21.3	0.00	0.00	0.95	0.04	* ONECUT1, * LOC101928499
		1518	chr17	59312894	61545486	17q23.2–q23.3	0.00	0.00	1.00	0.00	*EFCAB3*, *METTL2A*, * TLK2, * MRC2
Headache	T2D	76	chr1	153181186	154770139	1q21.3	0.04	0.00	0.94	0.02	* AQP10, * ATP8B2
		324	chr3	70449145	72528844	3p13	0.01	0.00	0.93	0.05	-
		564 ^a^	chr5	73759326	75797683	5q13.3	0.02	0.00	0.98	0.01	*HMGCR*, *COL4A3BP*, *POLK*, * ANKDD1B, * POC5
		745	chr7	1353067	2062006	7p22.3	0.00	0.00	0.97	0.03	* MAD1L1
		1370 ^a^	chr14	94325596	95750857	14q32.12–q32.13	0.00	0.00	1.00	0.00	*SERPINA2*, * SERPINA1

T2D: Type 2 diabetes; Chr: chromosome; Start bp: Start base pair; End bp: End base pair; PPA1: posterior probability for model 1 (association only to trait 1); PPA2: posterior probability for model 2 (association only to trait 2); PPA3: posterior probability for model 3 (shared association to both traits); PPA4: posterior probability for model 4 (two distinct associations of both trait). ^a^ Genomic regions are common to both migraine and T2D, and headache and T2D. * Genes have top SNP with single-trait *p* value < 1 × 10^−5^ for migraine, headache, and T2D.

**Table 3:** Novel LD-independent (*r*^2^ < 0.1) lead SNPs from cross-trait meta-analysis of migraine and T2D GWAS (*P*_meta_ < 5 × 10^−8^; single-trait FDR-adjusted *p* < 0.05).

Lead SNP	CHR	BP	EA	NEA	FE Meta-Analysis		Migraine			T2D			Variant Annotation	Nearest Coding Gene
					OR	*p*-Value	OR	*p*-Value	FDR	OR	*p*-Value	FDR		
rs11590235	1	2208123	T	C	1.05	4.33 × 10^−9^	1.05	3.59 × 10^−6^	1.77 × 10^−3^	1.05	3.00 × 10^−4^	3.22 × 10^−2^	Intronic	*SKI*
rs1841499	1	72836456	A	T	0.98	2.86 × 10^−8^	0.98	2.64 × 10^−4^	3.78 × 10^−2^	0.97	1.65 × 10^−5^	4.03 × 10^−3^	Intergenic	*NEGR1*
rs6748072	2	202980887	A	G	0.98	3.00 × 10^−8^	0.98	7.20 × 10^−5^	1.62 × 10^−2^	0.98	9.37 × 10^−5^	1.45 × 10^−2^	Non-coding transcript exon	*KIAA2012*
rs9817547	3	18753414	C	A	0.98	1.88 × 10^−8^	0.98	5.34 × 10^−5^	1.31 × 10^−2^	0.97	8.02 × 10^−5^	1.30 × 10^−2^	Intronic	*SATB1*
rs536445	3	173120103	C	T	0.98	1.35 × 10^−9^	0.98	2.07 × 10^−5^	6.66 × 10^−3^	0.97	1.21 × 10^−5^	2.85 × 10^−3^	Intronic	* *NLGN1*
rs4619890	4	7853160	G	A	1.02	2.84 × 10^−9^	1.03	7.54 × 10^−7^	5.30 × 10^−4^	1.02	8.58 × 10^−4^	4.79 × 10^−2^	Intronic	*AFAP1*
rs6829081	4	48693247	T	A	0.97	1.17 × 10^−10^	0.98	3.89 × 10^−5^	1.05 × 10^−2^	0.96	2.71 × 10^−7^	1.18 × 10^−4^	Intronic	*FRYL*
rs171697	5	103956516	G	C	1.03	7.73 × 10^−10^	1.03	7.93 × 10^−7^	5.52 × 10^−4^	1.03	2.36 × 10^−4^	2.74 × 10^−2^	Intronic	*NUDT12*
rs29648	5	170559580	A	G	0.98	3.10 × 10^−8^	0.98	3.26 × 10^−5^	9.30 × 10^−3^	0.97	2.50 × 10^−4^	2.27 × 10^−2^	Intronic	*TLX3*
rs62442924	7	1989976	T	C	0.97	1.94 × 10^−8^	0.98	3.33 × 10^−4^	4.37 × 10^−2^	0.96	6.80 × 10^−6^	1.06 × 10^−3^	Intronic	* *ELFN1*
rs6947337	7	41854681	A	G	0.98	3.90 × 10^−8^	0.98	7.78 × 10^−5^	1.70 × 10^−2^	0.98	1.20 × 10^−4^	1.60 × 10^−2^	Intergenic	*INHBA*
rs10101067	8	72407374	C	G	1.05	9.71 × 10^−10^	1.04	1.40 × 10^−5^	5.00 × 10^−3^	1.05	1.47 × 10^−5^	4.15 × 10^−3^	Intronic	*EYA1*
rs11140324	9	86634309	T	C	0.97	1.65 × 10^−9^	0.97	7.51 × 10^−6^	3.09 × 10^−3^	0.97	5.11 × 10^−5^	7.49 × 10^−3^	Intergenic	*RMI1*
rs2670139	9	126634255	C	T	1.03	3.11 × 10^−12^	1.03	1.43 × 10^−6^	8.71 × 10^−4^	1.04	2.82 × 10^−7^	1.24 × 10^−4^	Intronic	*DENND1A*
rs72854192	11	9587144	T	A	1.06	3.52 × 10^−8^	1.06	2.27 × 10^−5^	7.14 × 10^−3^	1.06	4.16 × 10^−4^	3.07 × 10^−2^	Intergenic	*WEE1*
rs11233452	11	82796110	G	A	1.03	9.52 × 10^−9^	1.02	1.32 × 10^−4^	2.45 × 10^−2^	1.03	1.08 × 10^−5^	2.15 × 10^−3^	Intronic	*RAB30*
rs10875762	12	48580759	G	A	1.03	1.83 × 10^−9^	1.02	8.09 × 10^−5^	1.75 × 10^−2^	1.04	3.06 × 10^−6^	7.87 × 10^−4^	Downstream	*CCDC184*
rs116862713	12	120185393	T	C	1.07	1.01 × 10^−8^	1.06	8.87 × 10^−5^	1.86 × 10^−2^	1.08	2.42 × 10^−5^	5.46 × 10^−3^	Intronic	*PRKAB1*
rs4902684	14	69445385	T	G	1.03	1.56 × 10^−10^	1.02	2.81 × 10^−5^	8.37 × 10^−3^	1.04	5.73 × 10^−7^	2.55 × 10^−4^	5′ UTR	*ACTN1*
rs299717	18	46163555	T	C	1.03	3.98 × 10^−8^	1.03	3.62 × 10^−4^	4.60 × 10^−2^	1.04	1.59 × 10^−5^	2.29 × 10^−3^	Intronic	*CTIF*
rs1013710	20	39882781	A	G	1.02	7.42 × 10^−9^	1.02	3.25 × 10^−6^	1.64 × 10^−3^	1.02	5.87 × 10^−4^	4.86 × 10^−2^	Intronic	*ZHX3*
rs4809370	20	62470872	T	C	0.98	1.06 × 10^−8^	0.98	1.34 × 10^−4^	2.47 × 10^−2^	0.97	1.50 × 10^−5^	2.85 × 10^−3^	Downstream	*ZBTB46*
rs28457031	22	41597228	A	G	1.07	9.99 × 10^−9^	1.07	1.10 × 10^−5^	4.17 × 10^−3^	1.07	2.29 × 10^−4^	1.67 × 10^−2^	Upstream	*L3MBTL2*

SNP: Single nucleotide polymorphism; CHR: Chromosome; BP: Base pair position (NCBI37/hg19); EA: Effect allele; NEA: Non-effect allele; OR: Odds ratio for EA; FE: Fixed effect; FDR: FDR-adjusted *p* value; T2D: Type 2 Diabetes, * These mapped genes are common with the genes found in cross-trait meta-analysis of headache and T2D.

**Table 4:** Novel LD-independent (*r*^2^ < 0.1) lead SNPs from cross-trait meta-analysis of headache and T2D GWAS (*P*_meta_ < 5 × 10^−8^; single-trait FDR-adjusted *p* < 0.05).

Lead SNP	CHR	BP	EA	NEA	FE Meta-Analysis		Headache			T2D			Variant Annotation	Nearest Coding Gene
					OR	*p*-Value	OR	*p*-Value	FDR	OR	*p*-Value	FDR		
rs546738	3	173117548	G	T	1.03	3.38 × 10^−10^	1.03	6.48 × 10^−6^	7.69 × 10^−3^	1.03	1.21 × 10^−5^	3.26 × 10^−3^	Non-coding transcript exon	*NLGN1*
rs73050128	7	1961882	A	C	0.96	1.50 × 10^−11^	0.96	3.04 × 10^−7^	5.53 × 10^−4^	0.96	1.06 × 10^−5^	2.87 × 10^−3^	Intronic	*ELFN1*
rs12432645	14	69599483	T	G	1.03	8.47 × 10^−10^	1.03	1.21 × 10^−5^	1.26 × 10^−2^	1.03	1.67 × 10^−5^	4.53 × 10^−3^	Intronic	*DCAF5*

SNP: Single nucleotide polymorphism; CHR: Chromosome; BP: Base pair position (NCBI37/hg19); EA: Effect allele; NEA: Non-effect allele; OR: Odds ratio for EA; FE: Fixed effect; FDR: FDR-adjusted *p* value; T2D: Type 2 Diabetes.

**Table 5:** Results for independent gene-based and gene-level genetic overlap analysis between migraine and T2D.

	Discovery	Target	Number of Overlapping Genes between Migraine and T2D	Proportion of Overlapping Genes between Migraine and T2D		Binomial Test *p*-Value
**Trait**	**Migraine**	**T2D**		**Expected**	**Observed**	
**Total number of genes**						
Raw number of genes	18309	18309				
Effective number of independent genes	13757	13694				
**Genes with *p*-value ≤ 0.1**				Genes with *p*-value ≤ 0.1		
Raw number of genes	5965	7305	2786	0.367	0.474	1.29 × 10^−44^
Effective number of independent genes	4102	5023	1943			
Proportion of effective number of genes	0.298	0.367				
**Genes with *p*-value ≤ 0.05**				Genes with *p*-value ≤ 0.05		
Raw number of genes	4421	5677	1774	0.281	0.403	2.83 × 10^−46^
Effective number of independent genes	2959	3850	1193			
Proportion of effective number of genes	0.215	0.281				
**Genes with *p*-value ≤ 0.01**				Genes with *p*-value ≤ 0.01		
Raw number of genes	2236	3348	662	0.159	0.295	1.24 × 10^−37^
Effective number of independent genes	1391	2171	411			
Proportion of effective number of genes	0.101	0.159				

Migraine: Migraine 2022 GWAS data from IHGC; T2D: T2D 2018 GWAS data from DIAGRAM consortium; Expected: Expected proportion of overlapping genes between migraine and T2D; Observed: Observed proportion of overlapping genes between migraine and T2D.

**Table 6:** Results for independent gene-based and gene-level genetic overlap analysis between headache and T2D.

	Discovery	Target	Number of Overlapping Genes between Headache and T2D	Proportion of Overlapping Genes between Headache and T2D		Binomial Test *p*-Value
**Trait**	**Headache**	**T2D**		**Expected**	**Observed**	
**Total number of genes**						
Raw number of genes	18261	18261				
Effective number of independent genes	13002	13143				
**Genes with *p*-value ≤ 0.1**				Genes with *p*-value ≤ 0.1		
Raw number of genes	3993	7350	1844	0.380	0.477	5.99 × 10^−25^
Effective number of independent genes	2719	4997	1297			
Proportion of effective number of genes	0.209	0.380				
**Genes with *p*-value ≤ 0.05**				Genes with *p*-value ≤ 0.05		
Raw number of genes	2659	5612	1050	0.287	0.412	4.08 × 10^−29^
Effective number of independent genes	1731	3770	714			
Proportion of effective number of genes	0.133	0.287				
**Genes with *p*-value ≤ 0.01**				Genes with *p*-value ≤ 0.01		
Raw number of genes	1163	3257	353	0.161	0.325	6.36 × 10^−25^
Effective number of independent genes	653	2119	212			
Proportion of effective number of genes	0.050	0.161				

Headache: Headache GWAS data from UK Biobank Neale lab; T2D: T2D data from DIAGRAM consortium; Expected: Expected proportion of overlapping genes between headache and T2D; Observed: Observed proportion of overlapping genes between headache and T2D.

**Table 7:** Genome-wide significant genes for migraine and T2D.

Genes	Chr	Start Position (hg19)	End Position (hg19)	LD Relationship between Top SNPs (*r*^2^)	Migraine			T2D		
					Gene *p*-Value	Top SNP	Top SNP *p*-Value	Gene *p*-Value	Top SNP	Top SNP *p*-Value
*MACF1*	1	39549839	39952810	0.920	2.76 × 10^−6^	rs1472662	1.75 × 10^−8^	1.25 × 10^−22^	rs61779275	7.90 × 10^−25^
*KIAA0754*	1	39875176	39882154	1.000	1.04 × 10^−6^	rs113214136	8.05 × 10^−8^	2.18 × 10^−23^	rs113214136	2.30 × 10^−24^
*BMP8A*	1	39957318	39995541	0.703	2.74 × 10^−6^	rs61779314	9.60 × 10^−8^	1.51 × 10^−23^	rs72663520	3.10 × 10^−25^
*THADA*	2	43457975	43823185	0.062	6.29 × 10^−8^	rs12712881	3.50 × 10^−10^	4.13 × 10^−28^	rs80147536	2.70 × 10^−30^
*SLC9B1*	4	103806205	103947552	0.520	3.47 × 10^−6^	rs4645215	9.53 × 10^−8^	2.34 × 10^−7^	rs13150953	6.70 × 10^−9^
*ANKDD1B*	5	74907301	74967671	0.499	4.80 × 10^−11^	rs42854	9.39 × 10^−13^	2.27 × 10^−12^	rs34341	5.70 × 10^−14^
*POC5*	5	74970023	75013313	0.650	4.20 × 10^−11^	rs42854	9.39 × 10^−13^	1.48 × 10^−14^	rs2307111	3.30 × 10^−16^
*NEU1*	6	31826829	31830709	0.010	1.38 × 10^−7^	rs41267082	5.50 × 10^−9^	6.02 × 10^−9^	rs9267653	2.40 × 10^−10^
* SLC44A4	6	31830969	31846823	0.005	1.72 × 10^−7^	rs74434374	4.51 × 10^−9^	4.58 × 10^−14^	rs9267658	1.20 × 10^−15^
* EHMT2	6	31847536	31865464	0.005	1.59 × 10^−7^	rs74434374	4.51 × 10^−9^	4.22 × 10^−14^	rs9267658	1.20 × 10^−15^
* PLEKHA1	10	124134094	124191871	0.070	2.78 × 10^−7^	rs76568359	6.38 × 10^−9^	8.73 × 10^−12^	rs2280141	2.00 × 10^−13^
*CALCB*	11	15095143	15103888	0.011	4.56 × 10^−7^	rs10741662	2.16 × 10^−8^	7.82 × 10^−7^	rs74643981	3.70 × 10^−8^
*CELF1*	11	47487489	47574792	1.000	9.73 × 10^−7^	rs7124681	2.15 × 10^−8^	2.90 × 10^−7^	rs7124681	6.40 × 10^−9^
*PTPMT1*	11	47586888	47595013	1.000	3.77 × 10^−7^	rs12798028	2.86 × 10^−8^	1.21 × 10^−7^	rs12798028	9.20 × 10^−9^
*KBTBD4*	11	47593749	47600567	1.000	4.50 × 10^−7^	rs12798028	2.86 × 10^−8^	1.45 × 10^−7^	rs12798028	9.20 × 10^−9^
*NDUFS3*	11	47600562	47606115	1.000	3.06 × 10^−7^	rs11039307	1.84 × 10^−8^	1.46 × 10^−7^	rs12798028	9.20 × 10^−9^
*FAM180B*	11	47608230	47610746	1.000	3.36 × 10^−7^	rs11039307	1.84 × 10^−8^	1.60 × 10^−7^	rs12798028	9.20 × 10^−9^
*C1QTNF4*	11	47611216	47615961	1.000	3.26 × 10^−7^	rs11039307	1.84 × 10^−8^	1.55 × 10^−7^	rs12798028	9.20 × 10^−9^
*MTCH2*	11	47638858	47664206	1.000	1.11 × 10^−7^	rs12419507	4.53 × 10^−9^	7.36 × 10^−7^	rs11039324	3.00 × 10^−8^
*PSMA3*	14	58711523	58738727	0.523	2.71 × 10^−7^	rs9323331	8.89 × 10^−9^	2.59 × 10^−6^	rs12892257	8.50 × 10^−8^
* BCAR1	16	75262928	75301951	0.084	7.91 × 10^−8^	rs2865826	1.07 × 10^−9^	8.87 × 10^−22^	rs72802395	1.20 × 10^−23^
* CFDP1	16	75327608	75467387	0.001	1.46 × 10^−11^	rs34624768	1.71 × 10^−13^	8.56 × 10^−13^	rs72804157	1.00 × 10^−14^
* TMEM170A	16	75477136	75498584	0.142	5.74 × 10^−11^	rs1030261	1.38 × 10^−12^	2.20 × 10^−9^	rs56258397	5.30 × 10^−11^
* CHST6	16	75507022	75528926	0.088	2.86 × 10^−7^	rs12924333	4.95 × 10^−9^	6.94 × 10^−8^	rs72789426	1.20 × 10^−9^
*SUGP1*	19	19387320	19431321	0.003	5.01 × 10^−7^	rs74182632	1.43 × 10^−8^	2.94 × 10^−13^	rs8107974	6.30 × 10^−15^
*MAU2*	19	19431496	19469563	0.003	4.55 × 10^−7^	rs34351431	1.48 × 10^−8^	1.10 × 10^−11^	rs73001065	3.00 × 10^−13^
*GATAD2A*	19	19496642	19619741	0.002	1.95 × 10^−6^	rs113920263	3.26 × 10^−8^	2.69 × 10^−10^	rs3794991	4.50 × 10^−12^
*TSSK6*	19	19625028	19626469	0.215	8.70 × 10^−7^	rs34183201	5.24 × 10^−8^	1.83 × 10^−7^	rs7252888	1.10 × 10^−8^
*NDUFA13*	19	19626550	19639858	0.209	5.61 × 10^−7^	rs34539063	2.70 × 10^−8^	2.29 × 10^−7^	rs7252888	1.10 × 10^−8^
*CILP2*	19	19649057	19657468	0.003	6.41 × 10^−7^	rs34539063	2.70× 10^−8^	2.37 × 10^−9^	rs17216525	1.00 × 10^−10^
*LPAR2*	19	19734464	19739039	0.003	3.03 × 10^−6^	rs2304129	1.73 × 10^−7^	2.98 × 10^−9^	rs73004975	1.70 × 10^−10^
*EYA2*	20	45523263	45817492	0.005	1.56 × 10^−6^	rs6124969	7.81 × 10^−9^	1.67 × 10^−8^	rs6063048	5.80 × 10^−11^
*L3MBTL2*	22	41601312	41627276	0.243	1.99 × 10^−6^	rs5751069	6.92 × 10^−8^	2.86 × 10^−6^	rs2038209	1.20 × 10^−7^

Chr: Chromosome, T2D: Type 2 Diabetes, hg19: human genome version 19, Genes: RefSeq genes, * These genes are common with the genome-wide significant genes found in headache and T2D.

**Table 8:** Genome-wide significant genes for headache and T2D.

Gene	Chr	Start Position (hg19)	End Position (hg19)	LD Relationship between Top SNPs (*r*^2^)	Headache			T2D		
					Gene *p*-Value	Top SNP	Top SNP *p*-Value	Gene *p*-Value	Top SNP	Top SNP *p*-Value
*HLA-C*	6	31236526	31239913	0.06	3.11 × 10^−6^	rs9264490	1.66 × 10^−7^	1.73 × 10^−9^	rs9264533	9.00 × 10^−11^
*SLC44A4*	6	31830969	31846823	0.03	3.77 × 10^−6^	rs652888	9.05 × 10^−8^	5.00 × 10^−14^	rs9267658	1.20 × 10^−15^
*EHMT2*	6	31847536	31865464	0.03	3.77 × 10^−6^	rs652888	9.05 × 10^−8^	4.99 × 10^−14^	rs9267658	1.20 × 10^−15^
*CYP21A2*	6	32006093	32009447	0.00	2.74 × 10^−6^	rs433061	1.52 × 10^−7^	1.01 × 10^−6^	rs115521560	5.60 × 10^−8^
*ATF6B*	6	32083045	32096017	0.01	1.71 × 10^−6^	rs1269852	7.46 × 10^−8^	4.81 × 10^−14^	rs3130342	2.10 × 10^−15^
*PLEKHA1*	10	124134094	124191871	0.07	2.66 × 10^−9^	rs78438709	5.37 × 10^−11^	1.04 × 10^−11^	rs2280141	2.00 × 10^−13^
*BCAR1*	16	75262928	75301951	0.08	4.79 × 10^−7^	rs12928974	6.04 × 10^−9^	9.52 × 10^−22^	rs72802395	1.20 × 10^−23^
*CFDP1*	16	75327608	75467387	0.11	2.38 × 10^−9^	rs3863442	2.48 × 10^−11^	1.05 × 10^−12^	rs72804157	1.00 × 10^−14^
*TMEM170A*	16	75477136	75498584	0.14	5.40 × 10^−9^	rs7198873	1.28 × 10^−10^	2.68 × 10^−9^	rs56258397	5.30 × 10^−11^
*CHST6*	16	75507022	75528926	0.09	2.65 × 10^−7^	rs12446877	3.88 × 10^−9^	8.20 × 10^−8^	rs72789426	1.20 × 10^−9^

Chr: Chromosome, T2D: Type 2 Diabetes, hg19: human genome version 19, Genes: RefSeq genes, Top SNP: Smallest *p*-value SNP associated with each gene.

**Table 9:** Results of the Mendelian randomisation analyses between migraine and headache with T2D using combined and sex-stratified summary-level GWAS data.

Exposure	Outcome	nSNPs	IVW		Weighted Median		MR-Egger		MR-PRESSO			nSNPs	GSMR	
			OR (95% CI)	*p*	OR (95% CI)	*p*	OR (95% CI)	*p*	OR	*p*	Global *p*		OR (95% CI)	*p*
T2D	Migraine	195	0.98 (0.96–1.00)	0.1	0.99 (0.97–1.02)	0.47	0.96 (0.91–1.02)	0.18	0.98	0.11	<2 × 10^−5^	314	0.97 (0.96–0.98)	9.9 × 10^−7^
T2D	Headache	195	0.98 (0.96–1.00)	0.124	0.98 (0.95–1.01)	0.206	0.96 (0.92–1.01)	0.119	0.99	0.19	<2 × 10^−5^	321	0.98 (0.97–1.00)	0.01
Migraine	T2D	96	0.98 (0.91–1.05)	0.59	0.96 (0.92–1.02)	0.18	0.87 (0.73–1.05)	0.16	1	0.89	<2 × 10^−5^	117	0.99 (0.96–1.02)	0.342
Headache	T2D	30	0.90 (0.84–0.97)	0.007	0.90 (0.83–0.97)	0.008	0.77 (0.62–0.97)	0.035	0.9	7 × 10^−5^	4.7 × 10^−3^	35	0.90 (0.84–0.95)	3.3 × 10^−5^
T2D (M)	Migraine (M)	19	1.02 (0.91–1.14)	0.743	0.96 (0.81–1.12)	0.592	0.97 (0.73–1.29)	0.825	1.02	0.71	0.80	29	1.02 (0.92–1.12)	0.69
T2D (M)	Headache (M)	33	0.97 (0.93–1.00)	0.072	0.99 (0.94–1.04)	0.711	0.93 (0.85–1.03)	0.18	0.97	0.12	0.02	50	0.99 (0.96–1.02)	0.43
Migraine (M)	T2D (M)	6	1.04 (0.93–1.15)	0.499	1.01 (0.90–1.14)	0.809	0.98 (0.46–2.07)	0.958	1.04	0.53	0.19	na *	-	-
Headache (M)	T2D (M)	6	0.84 (0.71–0.99)	0.049	0.83 (0.68–1.01)	0.062	0.64 (0.18–2.21)	0.519	0.84	0.007	0.96	74 *	0.97 (0.90–1.04)	0.37
T2D (F)	Migraine (F)	6	0.93 (0.85–1.02)	0.117	0.96 (0.86–1.08)	0.496	1.45 (0.58–3.64)	0.464	0.93	0.076	0.78	16	0.93 (0.88–0.98)	5.0 × 10^−3^
T2D (F)	Headache (F)	16	0.95 (0.92–0.98)	0.001	0.97 (0.93–1.01)	0.159	0.92 (0.85–1.00)	0.066	0.95	0.004	0.11	27	0.97 (0.95–0.99)	4.0 × 10^−3^
Migraine (F)	T2D (F)	6	0.95 (0.74–1.21)	0.666	0.96 (0.77–1.20)	0.701	0.63 (0.12–3.34)	0.613	0.95	0.68	0.07	27 *	0.97 (0.97–1.17)	0.21
Headache (F)	T2D (F)	21	1.04 (0.87–1.24)	0.701	1.11 (0.90–1.37)	0.343	1.15 (0.56–2.37)	0.7	1.04	0.705	0.14	22	0.98 (0.83–1.13)	0.8

IVW, Inverse variance weighted; MR Egger, Egger regression approach; MRPRESSO, Mendelian randomization pleiotropy residual sum and outlier; GSMR, Generalized summary data-based Mendelian randomization; nSNPs, Total number of SNPs used as genetic instruments; na, Number of SNPs was not sufficient for the GSMR analysis; OR, Odds ratio; CI, Confidence interval; M, Male; F, Female, Global *p*, Global test *p*-value; * *p* < 1 × 10^−5^ threshold used to extract genetic instruments.

## Data Availability

The data from the DIAGRAM consortium (https://www.diagram-consortium.org (accessed on 10 August 2019)) and the UK Biobank Neale lab are available on their respective websites. The full GWAS summary statistics for the 23andMe discovery data set will be made available through 23andMe to qualified researchers under an agreement with 23andMe that protects the privacy of the 23andMe participants. For further details and to access the data, please visit https://research.23andme.com/collaborate/#dataset-access/ (accessed on 11 December 2019). These data can be obtained by qualified researchers under an agreement with 23andMe that protects the privacy of the 23andMe participant. Researchers can perform a meta-analysis of 23andMe migraine summary statistics and IHGC migraine GWAS summary statistics to get the full summary statistics of the migraine 2022 GWAS dataset.

## Supplementary Materials

The following supporting information can be downloaded at: https://www.mdpi.com/article/10.3390/genes13101845/s1, Table S1: Summary of GWAS data, Table S2: Combined and sex-stratified SECA results for the test of genetic concordance between migraine and headache with T2D, Table S3: SNP-based single trait heritability between migraine and headache with T2D, Table S4: Shared pleiotropic regions between migraine and headache with T2D (PPA3 > 0.5), Table S5: Novel lead SNPs by cross-trait meta-analysis between Migraine and T2D GWAS, Table S6: Novel lead SNPs by cross-trait meta-analysis between Headache and T2D GWAS, Table S7: Validation of cross-trait meta-analysis approach, Table S8: Sex-stratified gene-based association analyses between migraine and headache with T2D, Table S9a: Genome-wide significant genes associated with migraine with their corresponding *p*-value in T2D genes in gene-based analysis, Table S9b: Genome-wide significant genes associated with headache with their corresponding *p*-value in T2D genes in gene-based analysis, Table S10: Genome-wide significant overlapping genes between migraine and T2D following gene-based analysis, Table S11: Genome-wide significant overlapping genes between headache and T2D following gene-based analysis, Table S12: Estimates of Genetic causality proportion (GCP) for Migraine and Headache with T2D by LCV, Table S13: Specific characteristics of genetic instruments of T2D phenotype on migraine and headache in 2SMR analysis, Table S14: Specific characteristics of genetic instruments of migraine and headache phenotype on T2D in 2SMR analysis, Table S15: Specific characteristics of genetic instruments of migraine and T2D used in GSMR analysis, Table S16: Specific characteristics of genetic instruments of headache and T2D used in GSMR analysis, Table S17: Pathways analysis for Migraine and T2D, Table S18: Pathways analysis for Headache and T2D, Table S19: Pathways analysis for Headache and T2D in Female, Table S20: Pathways analysis for Headache and T2D in Male.
